# Treatment of Chronic Back and Chest Pain in a Patient with Sickle Cell Disease Using Spinal Cord Stimulation

**DOI:** 10.4274/tjh.2017.0447

**Published:** 2018-11-13

**Authors:** Damla Yürük, İbrahim Aşık

**Affiliations:** 1 Bursa Yüksek İhtisas Training and Research Hospital, Clinic of Algology, Bursa, Turkey; 2 Ankara University Faculty of Medicine, Department of Algology, Bursa, Turkey

**Keywords:** Chronic pain, Sickle cell disease, Spinal cord stimulation

## To the Editor,

Pain with sickle cell disease can occur in two forms: acute or chronic. Acute pain is often treated with analgesics in emergency services or at home and can intermittently relapse. In the later stages of the disease, chronic pain occurs due to central sensitization. Here we report a patient with sickle cell pain who was treated with a spinal cord stimulator (SCS).

**Figure 1:**
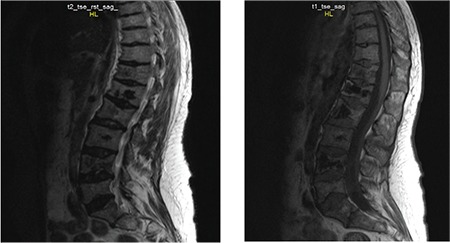
Magnetic resonance imaging shows multiple infarcts involving vertebral column T2 and T1 sagittal images. Non-enhancing areas involving vertebral bodies at dorsal and lumbar levels suggest infarcts.

Our patient was a 28-year-old female. She was admitted to the hospital due to painful crises and had a history of operations due to vertebral fracture, femoral head osteonecrosis, and pulmonary hypertension. Her back and chest pain was ranked as 9 on a numeric rating scale when she was referred to the pain clinic. Non-enhancing areas involving vertebral bodies at dorsal and lumbar levels, suggestive of infarcts, were shown by magnetic resonance imaging (Figure 1). Pain control could not be achieved medically; all nonsteroidal anti-inflammatory drugs and opioids had been unsuccessful. After evaluation by the local pain council of the hospital, the patient underwent implantation of an SCS.

**Figure 2:**
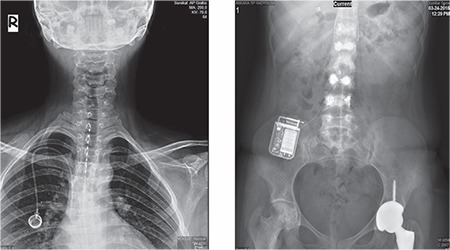
Plain X-ray demonstrating placement of spinal cord stimulator electrodes at the level of T1-T4.

In the operating room she was placed in the prone position. Under fluoroscopic guidance a 15-gauge Tuohy needle was inserted into the T6-T7 interlaminar space. Eight-electrode leads were inserted through the needle and advanced until the tip lay at the T1-T4 epidural level (Figure 2). Parameters of stimulation were pulse width of amplitude 2.5 mA and frequency of 10 kHz. Following more than 60% pain relief throughout the trial period, the lead was connected to an implantable pulse generator, which was placed into the left buttock. During 1-year follow-up of the implantation of the high-frequency SCS, excellent pain relief continued with improvement of both the patient’s pain and her ability to perform activities of daily living.

SCS has become popular in recent years. It is a neuromodulator and manages cases of certain chronic pain for which other procedures have failed, including failed back syndrome [1], ischemic limb pain [2], angina pectoris [3], and painful peripheral neuropathies [4]. The mechanism of pain relief by the SCS is still not clear [5]. According to the gate control theory, in the peripheral nerve system the afferent activity of large fibers or small fibers is controlled by the dorsal column cells, associated with central transmission of pain. When a surplus of large-fiber activity occurs, this gate closes [6]. The pathophysiology of sickle cell disease could also be related to autonomic nervous system efficiency [7]. Constriction or obstruction of a blood vessel usually causes a reduction in blood flow and oxygen delivery to the tissues and thus insufficient perfusion [8]. Small fibers, which carry nociceptive information in the sympathetic pain pathway, can be blocked by a high-frequency SCS.

The present report describes the first patient with intractable pain due to sickle cell disease who was treated with a high-frequency SCS successfully. For patients in whom  all available treatments have failed or who have an increased risk for more invasive surgical interventions, the SCS might be a therapeutic alternative.
